# An “all-wheel drive” proposal to accelerate clinical research in common and rare neurological diseases

**DOI:** 10.1007/s10072-019-04189-4

**Published:** 2019-12-19

**Authors:** Marco Salvetti, Mario A. Battaglia, Massimiliano Di Filippo, Gian Luigi Mancardi, Michelangelo Mancuso, Francesco Patti, Maria Pia Sormani, Paola Zaratin

**Affiliations:** 1 Department of Neuroscience Mental Health and Sensory Organs (NEMOS), Sapienza University, Sant’ Andrea Hospital, Rome, Italy; 2 IRCCS NEUROMED - Mediterranean Neurological Institute, Pozzilli, Italy; 3 Italian Multiple Sclerosis Foundation, Genoa, Italy; 4 Department of Life Sciences, University of Siena, Siena, Italy; 5 Section of Neurology, Department of Medicine, Santa Maria Della Misericordia Hospital, University of Perugia, 06132 Perugia, Italy; 6 Department of Neuroscience, Rehabilitation, Ophthalmology, Genetics, Maternal and Child Health, University of Genova and Scientific Clinical Institutes Maugeri IRCCS, Pavia-Genova Nervi, Italy; 7 Department of Clinical and Experimental Medicine, Neurological Institute, University of Pisa, Pisa, Italy; 8 Department of Medical and Surgical Sciences, and Advanced Technologies, “G.F. Ingrassia”, Multiple Sclerosis Center, University of Catania, Catania, Italy; 9 Department of Health Sciences, University of Genoa, Genoa, Italy

**Keywords:** Multiple sclerosis, Rare diseases, Pathophysiology, Clinical research, Therapies

## Abstract

The complex biology of neurological diseases calls for collaborative efforts that may increase the success rate of clinical research. Models have been proposed, but concrete actions remain insufficient. Based on recent considerations from basic science, from science of patient input and from an analysis of scientific resources in Italy, we here explain why our country may represent an appropriate environment for such actions. Furthermore, we sketch operational framework and business model to be applied in order to accelerate, in parallel, the development of therapies in common and rare diseases.

## Introduction

Neurological diseases pose a major and increasing burden on global health, which may even be underestimated if measured only by mortality [1]. The complex biology behind these conditions, and their profound social implications, suggests that a crucial piece to solve this puzzle will be achieving effective collaboration between different actors, e.g. academia, industry and patient-advocacy organizations. This collaboration is intrinsically difficult as these stakeholders have to negotiate solutions to common problems while advocating for return of investments that differ from one another.

Even within each category of actors, some characteristics of neurological disorders do not favour collaborative work. From an epidemiological viewpoint, neurological conditions are quite heterogeneous, going from some of the most frequent to the rarest diseases known. As a result, the resources they catalyse are highly variable, with many disorders that are underserved as far as research, healthcare investments and advocacy are concerned. Neurological diseases are heterogeneous also from a clinical perspective. Central and peripheral nervous system involvement, acute and chronic course and paediatric and geriatric age of onset are just some of the extremes that neurological disorders embrace. These clinical distances, and the increasing complexity of the management of some of these diseases, are gradually separating the work of physicians and physician scientists into sub-specialities with poorly communicating areas of knowledge. As a result, opportunities for cross-fertilization between different research areas diminish as each one develops its own subculture (not to speak of the other drawback, viz. the increase in the probability of clinical errors). Along the same lines, advocacy organizations who, by definition, represent each one its own disease are not encouraged towards collaboration if science and clinical approaches push them in the opposite direction.

Others and we have recently proposed models to improve the collaboration between the key stakeholders in the arena: academia, industry and patient-advocacy organizations [2–4]. We suggested that, based on the growing genetic and neurobiological evidence for pathophysiologies that are shared in common across numerous neurological conditions [4], coordinated clinical research initiatives referring to conditions that share biological mechanisms should be encouraged. In particular, we showed how rare diseases may provide opportunities to better understand discrete pathophysiologies that, in more frequent diseases, overshadow one another. Reciprocally, developing therapies across rare and frequent diseases may help develop the economies of scales that may help the industrialisation of drug development in rare conditions. We also proposed this as a new objective that may ignite a new willingness of collaboration also among organizations advocating for different diseases.

These themes were discussed during a symposium held on these themes during the 2018 National Congress of the Italian Neurological Society (SIN). Based on the arguments that emerged, the Italian Multiple Sclerosis (MS) Society, under the auspices of SIN, aims at verifying existing conditions and new ideas to speed up an harmonized development of – and access to – sustainable therapies in MS and in rare diseases.

As a preparatory stage, we here look at the resources that the Italian neurological community, and Italian patient-advocacy organizations, may offer to implement such actions in our country. Based on the existing assets, we then examine the additional steps that, if taken within new and participatory models of decision-making, might contribute to the creation of a unique environment for the advancement of neurological clinical research in Italy.

## Present resources

The international level of Italian research is one of the highest, particularly if considered in comparison with the investment in research, which is one of the lowest [5]. Notably, this result has been achieved in an environment that has been, for years, less competitive than that in other nations. The public nature of the Italian educational and research system puts less pressure on researchers compared with other countries, in particular the USA. Hence, though the economic crisis and an increased attention to meritocracy are now fostering competition (with excellent results, as Italy registered one of the steepest increases in the percentage of articles in the world’s top 10% cited papers; ref. [6], the “publish or perish” mantra has had, and probably still has, a relatively reduced impact on the Italian research community. With less competition, collaboration should in principle be easier. There is no proof that this happened in Italy. However, some examples of strikingly effective collaboration can be made, at least outside the neurology field: the Italian Group for Adult Haematological Diseases (GIMEMA) is a collaboration between 150 haematology centres on the Italian territory. Established in 1982, every year more than 3000 patients are in clinical trials (at present more than 50) followed by GIMEMA’s centres and research physicians. Over the years, GIMEMA has directly contributed to some of the most relevant therapeutic successes in haematology, such as the increased life expectancy of patients with acute myeloid leukaemia or the cure of acute promyelocytic leukaemia, formerly one of the most lethal haematological diseases.

In the neurological field, there are no examples of such collaborations yet. However, the landscape is rich of first-level research and clinical institutions, and patient-advocacy organizations, which may multiply their impact if only they would feel more encouraged to devoid part of their efforts to collaborative initiatives. In almost every aspect of neurobiology and neuropharmacology, and in almost every category of frequent and rare neurological diseases, there are researchers that compete and often emerge at the international level. Listing all of them is not the purpose of this article. Suffice here to say that in diseases like MS, there are groups with competencies that, if aligned, may give rise to an ideal drug discovery pipeline.

Besides, there are collaborative efforts that may support the development of such a pipeline.

The Italian MS Register was promoted and funded by the Italian MS Foundation (FISM) in collaboration with the Italian MS clinical centres in 2014. Its objective was the creation of an organized nationwide database for “better defining the disease epidemiology, improving quality of care, and promoting research projects in high-priority areas”. The register is expanding, also thanks to the implementation of a data collection website (https://registroitalianosm.it) [7]. Besides research priorities that have already been identified, the Register could be instrumental for the generation of trial-ready cohorts. In addition, the Italian MS clinical centres, through the Italian MS Register, are contributing to the post-authorisation safety study (PASS). Regulators, pharma, and registries have jointly identified a format of collaboration on PASS for MS disease-modifying treatments (DMTs) to benefit society and patients [7].

The Italian Neuroimaging Network Initiative (INNI; www.inni-ms.org) is an MRI databank established with the major aim to determine and validate novel MRI biomarkers. The network involves centres and investigators with an internationally recognized expertise and is well positioned to provide new predictors and outcome measures that may improve the assessment of efficacy in clinical trials [8]. Greater focus on development and implementation of MRI biomarkers to monitor disease activities in MS will have important implication to increase access to innovative therapies.

The biobank of CRESM (http://www.nico.ottolenghi.unito.it/eng/Research/Research-Groups/Clinical-neurobiology), run by the Clinical Neurobiology Laboratory of the Azienda Ospedaliera Universitaria San Luigi Gonzaga, collects serum, plasma, cerebrospinal fluid, peripheral blood mononuclear cells, DNA and RNA from persons with MS and controls. Though limited to CRESM patients, the biobank assures strict collection criteria plus standardized and validated procedures that improve the reproducibility of biological measures, including those that may be used in clinical trials.

Italian patient-advocacy organizations are not inferior to their academic partners relatively to their impact and international stature.

Patient-advocacy organizations and foundation/charities play an instrumental role in the research and development process of their disease area of interest. During the past 10 years, the international role of patient-advocacy organizations in health research has drastically changed. In Italy, some of them are no longer merely distributors of grant money but have reinvented themselves as on-profit enterprises with new, creative business models [3]. This shift might also reflect a change in the staffing of medical nonprofit foundations from grant managers to experienced pharma and biotech executives. This more active role poses a challenge of striking the right balance in distributing research investments across the research portfolio and to invest outside the disease area of primary interest in order to accomplish maximum benefit for patients. In this scenario, patient-advocacy organizations and foundation/charities have started adopting strategies characterized by an increasing involvement in projects’ governance, development and advancement (project management) and by proactive decision-making about the projects that are funded (portfolio management). The common theme among the existing examples is that patient-advocacy organizations and foundation/charities now invest directly into research infrastructures, drug candidates and therapy development [7–11]; (https://clinicaltrials.gov/ct2/show/NCT01854957?term=uccelli&cond=multiple+sclerosis&rank=1;https://clinicaltrials.gov/ct2/show/NCT03269071). Along these lines, MITOCON, the advocacy organization of persons with mitochondrial diseases, together with the Clinical Centres taking care of the vast majority of patients in Italy, has funded the creation of the Italian database of mitochondrial diseases (MDs). This nationwide Italian collaborative network has developed a web-based registry of patients with MDs, containing more than 1700 patients, with both adulthood and childhood onset cases (accessible on line at https://www.mitochondrialdisease.it). However, in developing a national initiative, it is of the utmost importance not to forget a broader perspective, even just to overcome the difficulties that the epidemiology of rare diseases entails. Hence, this platform will be integrated in an international register in collaboration with Germany, the UK and France (sponsored by the E-Rare: https://www.genomit.eu/project). This effort has already provided several patients for clinical trials and has launched a strong collaboration with industry and other stakeholders to develop new therapies and clinical trial readiness.

This is an example of how coordination of the activities of funding agencies, academic scientists and clinicians, companies, regulatory bodies and patient advocacy organizations in partnerships with European Union can maximize the global impact of investments in rare diseases research. Not without reason, the European Union has put much effort into funding research on rare diseases, encouraging national funding organizations to collaborate together in the E-Rare program and setting up European Reference Networks for rare diseases. This is demonstrably contributing to accelerating progress at multiple levels, including faster diagnosis through enhanced discovery of genes, better understanding of natural history of several rare diseases through creation of common registries and boosting of innovative therapeutic approaches.

Other excellent examples of cooperation towards a common shared agenda at the international level include the Multiple Sclerosis International Federation (MSIF). This federation is leveraging on National MS Society (acting as MSIF lead agencies) to implement the international 2017–2021 strategic MS agenda. Italian MS Society is the MSIF lead agency for patient-reported outcome data sharing among clinical centres, as a priority area for research collaboration between MS organizations (http://www.msif.org/wp-content/uploads/2017/08/MSIF-Strategy-2017-2021-web.pdf).

## Future action items

The potential of these research groups and organizations is evident and has already produced important results. Needless to say that, if finalized towards collaborative efforts, all these and other competencies and projects would probably increase their impact. However, this may still not be enough to make the difference for prolonged periods of time without experiencing a “consortium fatigue” that is always a threat for this kind of initiatives [2]. Much of the “exhaustion” for actors involved in health research comes from working without a realistic hope of seeing their work fully verified. This is particularly true in many neurological conditions where chronic disease course, lack of satisfactory outcome measures and biomarkers make the assessment of efficacy of any given treatment more cumbersome than for the majority of non-neurological diseases. In this perspective, it is clear that creating the opportunity to rapidly verify the ideas with more agile trials would energize both basic science and clinical researchers, reinforcing their ability to teamwork, thus becoming an antidote to tiredness.

Other countries represent good examples of collaborative efforts in this context. In the UK, the National MS Society is supporting an expert consortium that has the objective to design and deliver an efficient clinical trials platform to speed up the development of new treatments for progressive MS. We here propose a similar initiative but transversal to different neurological diseases. As already suggested [3], this may represent a step forward: developing therapies across biologically similar diseases may refine our ability to capture the biological efficacy of a pharmacological agent. Consequently, it may de-risk industrial investments, provide results that are perceived as more reliable by regulatory authorities and help develop the economies of scales that may promote the industrialisation of rare disease therapy development. The endeavour is new, and not an easy one, as it necessitates an infrastructure to select targets, treatments and diseases while generating trial designs that suit disease course and biology. In particular, as far as trial design is concerned, new and more flexible methods are required, based on innovative approaches such as adaptive strategies in Bayesian frameworks [12]. To experiment with the design of clinical trials and to implement innovative designs with continuous Bayesian monitoring [13], a large effort is needed at the design stage, when a large amount of simulations must be run by expert statisticians, with specific competencies in Bayesian statistics. Hence the need for a dedicated team of statisticians with specific competencies devoted not only to designing new trials but also to making research on new trial strategies. Ideally, a dedicated infrastructure should be devoted to clinical trial design development. The infrastructure should be part of a broader operational framework (Table 1), working not only on biological, medicinal chemistry and other issues key to drug development but also on the identification of resources (including clinical centres that may guarantee efficient recruitment and, when appropriate, industrial partners) and on plans for the clinical/industrial development of the experimental treatments.

**Table 1:** Operational framework

**Task**	**Competencies required**
Target identification	Neurobiology, immunology
Treatment selection	Medicinal chemistry, bioinformatics, clinical neurology
Trial design	Biostatistics
Identification of participating centres, industrial partners	Scientific societies, industry representatives
Clinical/industrial development	Advocacy, industry representatives, regulatory authorities

Based on clinical and biological considerations and on feasibility issues, we think that the first coordinated attempt to develop new therapies across different diseases (and in so doing test the above operational framework) should be centred around mitochondrial alterations [14]. For obvious reasons, mitochondrial metabolism is key to the functioning of all the different CNS cellular components and may be influenced by cells of both the adaptive and innate immune system. These and other aspects, e.g. developments in mitochondrial pharmacology [15], qualify mitochondrial defects as a potential therapeutic target in MS and in other common neurological diseases. Based also on considerations about the relatively high prevalence of Leber’s hereditary optic neuropathy compared with other mitochondrial conditions, we plan testing our operational framework on MS and on this disease though other choices cannot be excluded at this stage.

## New business models to accelerate the development of therapies for common and rare neurological diseases

Accelerating therapies for patients with frequent and rare brain disorders, through a collaborative approach, based on shared pathophysiologies and targets, challenges our notion of good science as such in the field of drug development. To make this space attractive for all the stakeholders involved, a strategic and collaborative investment framework and new business models will be required.

This is in line with the mission of Responsible Research and Innovation (RRI) EU’s Horizon 2020 programme that encourages different stakeholders to work together during the whole research and innovation process, to keep it aligned with the values, needs and expectations of society and patients. The RRI programme challenges our notion of good science by arguing that excellence, validity and relevance are connected by engaging patients and society in the research continuum as key stakeholders with a decision-making role. In this context, the Italian MS Society coordinates a project that aims at building a framework to enable multi-stakeholder initiatives. Founding principles of the framework will be increasing result-based accountability and empowering the assessment of the social impact of the initiative, with special attention to how research affects patient lives (https://www.multiact.eu/). This project represents a timely opportunity and an important reference to explore ideas and establish conditions for a collaborative development of new therapies for MS and for rare diseases.
